# Evaluation of the efficacy and quality of life in patients with temporomandibular joint disorders treated with Kovacs digital occlusal splint: a pilot study

**DOI:** 10.1186/s12903-024-04572-4

**Published:** 2024-07-16

**Authors:** Qiang Xu, Jing Li, Chi Wang, Sun-Qiang Hu, Yin Chen, Xin Nie, Jin Xiao

**Affiliations:** Department of Oral Maxillofacial Surgery, School and Hospital of Stomatology, Wenzhou Medical University, No. 1288 Longyao Avenue, Longwan District, Wenzhou, 325000 Zhejiang China

**Keywords:** Occlusal splint, Temporomandibular disorders, Osteoarthritis, Logistic regression, Quality of life, Disease severity

## Abstract

**Background:**

Few studies have been conducted on treating temporomandibular disorders (TMDs) with new digital occlusal splints, which has increasingly attracted wide attention.

**Methods:**

To evaluate the clinical efficacy and quality of life (QoL) of Kovacs digital occlusal splint (KDOS) treatment in patients with TMD.

**Materials and methods:**

Eighty-nine patients with TMD who were treated using KDOS were analyzed. The patients were divided into three groups according to the Wilkes stage. The clinical symptoms and QoL scores of the patients in each group were recorded before and at least three months after treatment, and the data were statistically analyzed and compared. The relationships between the disease severity, sex, age, and level of QoL before treatment and improvement in the clinical symptoms were analyzed using binary logistic regression.

**Results:**

The mean age and follow-up period of the patients were 28.0 ± 10.4 years and 4.9 ± 2.1 months, respectively. After KDOS treatment, the improvement rates of joint noise and pain were 80.4% and 69.8%, respectively. Additionally, the patients’ maximum mouth opening and global QoL mean scores significantly improved compared to those before treatment (*p* < 0.001). Binary logistic regression analysis revealed that the factors affecting the improvement in the clinical symptoms were disease severity and level of QoL before treatment.

**Conclusions:**

KDOS can improve the clinical symptoms and QoL of patients with TMD. Moreover, patients without osteoarthritis and with low pretreatment QoL levels are more likely to demonstrate clinical improvement.

**Trial registration:**

The trial was registered with Chinese Clinical Trial Registry (ChiCTR) (ID: ChiCTR2300076518) on 11/10/2023.

## Background

Temporomandibular disorders (TMDs) do not comprise a single disease entity, it is a general term for diseases involving pain and dysfunction of the temporomandibular joint and masticatory muscles. The main symptoms are joint crackling, murmurs, joint pain, and jaw movement disorders [1]. TMDs are very common in the general population [2–4]. The prevalence of symptoms varies from 25 to 50% while the prevalence of clinical signs varies from 40 to 90% [5]. Previous studies on patient samples have indicated substantial negative effects of TMD on the oral health-related quality of life (OHRQoL) [6, 7]. The etiology of TMD is vast and unclear with various complicated theories, thereby affecting the establishment of a correct diagnosis [8, 9]. Since occlusal factors are the most dominant pathogenic factors of TMD, treatment using occlusal splints, which is widely used in clinical practice, has been recognized as an area of increasing interest among researchers [10, 11].

Currently, the stabilization splint is the most commonly used occlusal splint. However, owing to the complicated traditional manufacturing process and low degree of automation, ensuring accuracy of occlusal splints is often challenging. Many inconveniences of the traditional methods can be solved with the help of digital technology [12]. Kovacs digital occlusal splint (KDOS) is an innovation of traditional occlusal splint technology, which obtains ideal jaw position after muscle deprogramming, and customizes complete digital and comfortable occlusal splints for patients with TMD [13]. It can standardize the production process, thereby saving time by reducing the influence of human factors and patient visits.

Being a common disease, TMD affects many patients and impairs their QoL. KDOS, which is a new digital occlusal splint for TMD treatment, has generated great interest. The present study aimed to evaluate the clinical efficacy and QoL of patients with TMD using KDOS.

## Methods

**Fig. 1:**
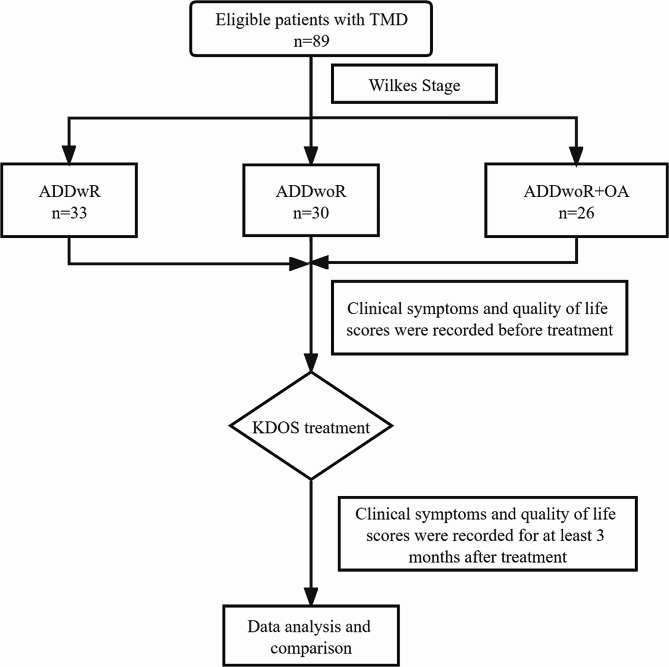
Research flow chart. Abbreviations: TMD, temporomandibular disorders; ADD, anterior disc displacement; wR, with reduction; woR, without reduction; OA, osteoarthritis; KDOS, Kovacs digital occlusal splint

This study was approved by the Ethics Committee of Wenzhou Medical University (WYKQ2022013); all the authors have read the Declaration of Helsinki, and each patient provided signed informed consent. The patients in this study were diagnosed with TMD and received KDOS treatment in our department between September 2022 and May 2023. The inclusion criteria for the study were as follows: (1) anterior disc displacement with or without reduction (ADDwR or ADDwoR) confirmed by magnetic resonance imaging, (2) age ≥ 18 years, (3) no history of any treatment for TMD, and (4) at least having a 3-month follow-up record. The exclusion criteria were as follows: (1) long-term chronic diseases, such as diabetes and immune diseases, which may affect the QoL of the patients; (2) cognitive dysfunction; and (3) incomplete follow-up data. The enrolled patients were divided into three groups based on the Wilkes–Bronstein classification: (1) ADDwR, (2) ADDwoR, and (3) ADDwoR + osteoarthritis (OA) groups. The period before and at least 3 months after treatment when the patients returned to the hospital for examination was selected as the time point, the relevant clinical symptoms were recorded by the same experienced doctor, and a trained specialist nurse guided the eligible patients to complete the questionnaire. Any concerns regarding the questionnaire guidelines were clarified, and the integrity of the completed questionnaire was assessed. The issuance, guidance, and collection of the questionnaires were conducted by the same nurses to ensure accuracy. The research flowchart is presented in Fig. 1.

### KDOS treatment

**Fig. 2:**
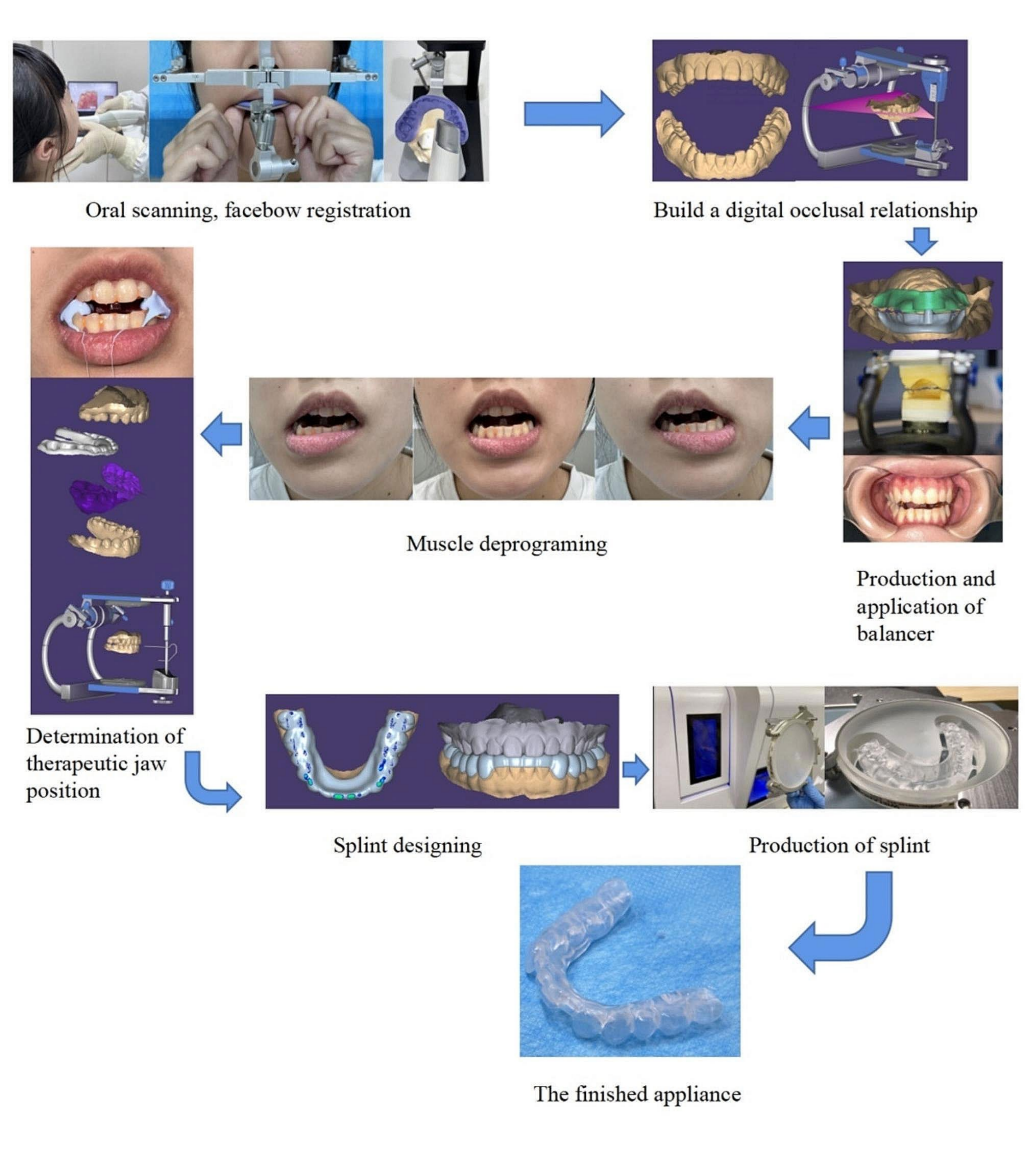
Kovacs digital occlusal splint production flow chart

Eligible patients were treated using KDOS. First, the dentition and occlusal relationship of the patient were scanned using an oral scanner (3shape, Copenhagen, Denmark), and a balancer was fabricated according to the digital data. Second, the stable therapeutic jaw position was recorded after wearing a balancer and performing muscle-deprogrammed movements, which were imported into the Exocad software (Version 3.0, Exocad GmbH, Darmstadt, Germany) for the design of the occlusal splint. Finally, a semi-anatomical occlusal splint made of resin discs (PMMA disk; Yamahachi Dental, Japan) was automatically ground using the SELECT five-axis engraving machine (Wieland, Germany) [13]. The KDOS production flow chart is shown in Fig. 2. All the clinical procedures were performed by an experienced physician. The patients were required to visit the doctor three times for KDOS treatment, and corresponding adjustments were made during the first week after wearing it. Follow-ups were conducted once every 1–2 months according to the condition of the patient.

### Clinical symptoms and questionnaire data collection

Before and at least three months after wearing KDOS, pain, joint noise, and maximal mouth opening (MMO) were measured using the pain-visual analog scale (pain-VAS), joint noise score (JNS) in the Friction Index, and straight edge, respectively. Pain-VAS scores from 0 to 10 and JNS scores from 0 to 4 represent low to high levels. MMO, the maximum distance at which the patients could open their mouth, was evaluated as the distance between the edges of the maxillary and mandibular incisors. The pain-VAS score and JNS were considered improved in cases of a decline by more than or equal to 30% after treatment. The improvement rates before and after treatment were compared. The Depression, Anxiety, and Stress Scale-21 (DASS-21) and Oral Health Impact Profile for TMDs (OHIP-TMD) were used to record the QoL of the patients before and after treatment. To measure and distinguish the symptoms of depression, anxiety, and stress, the DASS-21 consists of three sections, each with seven items, with responses ranging from 0 ( does not apply to me at all) to 3 (applicable to me very much or most of the time). The Chinese version has been validated across cultures [14]. The OHIP-TMD includes 22 items in seven domains, namely functional limitation, physical pain, psychological discomfort, physical disability, psychological disability, social disability, and handicap. Each item was assessed using a 5-point response scale as follows: 0 = never, 1 = hardly ever, 2 = sometimes, 3 = fairly often, and 4 = very often. Higher scores indicate worse QoL. The validated standard Chinese version of the OHIP-TMD was used according to the respective guidelines [15].

### Statistical analysis

SPSS Statistics version 27.0 software (IBM Corp., Armonk, NY, USA) was used for the data analysis. The Wilcoxon signed-rank test was used to analyze the changes in the questionnaire scores and MMO before and after treatment. The chi-square test was used to compare the improvement rates of the JNS and Pain-VAS scores before and after treatment. Binary logistic regression was used to analyze whether the sex, age, group, and pre-treatment QoL scores were associated with improved clinical outcomes.

## Results

Eighty-nine patients were finally enrolled in the study; 33, 30, and 26 patients were present in the ADDwR, ADDwoR, and ADDwoR + OA groups, respectively, which included 62 women and 27 men, with an average age of 28.0 ± 10.4 years and an average follow-up of 4.9 ± 2.1 months (Table 1). The overall age distribution ranges from 18 to 55 years old, with 42.7% of the respondents between 20 and 30 years old. Following KDOS treatment, the improvement rate of the ADDwR group was the highest, whereas that of the ADDwoR + OA group was the worst. The improvement rates of the JNS and Pain-VAS score were 80.4% and 69.8%, respectively, which were significantly improved compared with those before treatment (*p* < 0.001, Table 2). MMO also improved significantly (30.8 ± 6.6, 36.2 ± 5.7, *p* < 0.001, Table 2).

**Table 1:** Basic patient information

Index	ADDwR	ADDwoR	ADDwoR + OA	Total
Cases	33	30	26	89
Gender				
Male	10	8	9	27
Female	23	22	17	62
Age (year)	22.2 ± 9.3	28.1 ± 11.5	31.4 ± 7.4	26.9 ± 10.4
follow-up (months)	4.7 ± 2.0	5.4 ± 2.2	4.6 ± 1.9	4.9 ± 2.1

Abbreviations: ADD, anterior disc displacement; wR, with reduction; woR, without reduction; OA, osteoarthritis. The chi-square test was used to assess the significance of the data

**Table 2:** Changes of clinical symptoms in three groups of patients before and after treatment

Index	Cases before treatment	Cases after treatment	Improvement rate (%)	*p* value
JNS				
ADDwR	25	22	88	
ADDwoR	17	14	82	
ADDwoR + OA	9	5	67	
Global	51	41	80.4	*p*<0.001
Pain-VAS				
ADDwR	18	13	83	
ADDwoR	20	16	80	
ADDwoR + OA	15	8	53.3	
Global	53	37	69.8	*p*<0.001
MMO				
ADDwR	33.6 ± 6.4	37.0 ± 4.8	-	
ADDwoR	28.7 ± 5.1	36.1 ± 5.3	-	
ADDwoR + OA	29.7 ± 7.1	34.9 ± 6.8	-	
Global	30.8 ± 6.6	36.2 ± 5.7	-	*p*<0.001

Abbreviations: JNS, joint noise score; VAS, visual analogue scale; MMO, maximal mouth opening; ADD, anterior disc displacement; wR, with reduction; woR, without reduction; OA, osteoarthritis; The Kolmogorov-Smirnov (K-S) test is used to analyze whether the data conform to the normal distribution. The Wilcoxon signed-rank test and chi-square test were used to assess the significance of the data

### OHIP-TMD

Global OHIP scores before and after treatment fluctuated between 0 and 18 and 0–17, respectively. Most domain scores demonstrated an upward trend from the ADDwR to the ADDwoR + OA group. The mean global OHIP score following KDOS treatment was significantly lower than that before treatment (6.1 ± 3.2, 7.2 ± 3.7, respectively; *p* < 0.001). Significant differences in the mean scores before and after treatment were also observed in all the seven domains. After treatment with KDOS, a significant decrease in the scores in each domain was observed. Only the ADDwoR + OA group demonstrated no significant difference in the domain scores of physical disability and handicap before and after treatment (5.1 ± 1.5, 5.0 ± 1.6, *p* = 0.718; 4.8 ± 1.4, 4.4 ± 1.6, *p* = 0.058, respectively). The OHIP scores are presented in Table 3.

**Table 3:** OHIP-TMD scores in three groups before and after treatment

Domain	Before treatment					After treatment					*p* value
	Mean	SD	Median	Interquartile spacing	Range	Mean	SD	Median	Interquartile spacing	Range	
Functional limitation											
ADDwR	5.5	2.1	6	2.5	0–8	4.3	1.7	4	2	0–8	*p* < 0.001
ADDwoR	5.6	1.6	6	2	1–8	4.6	1.6	5	2	1–8	*p* < 0.001
ADDwoR + OA	5.9	1.3	6	2	3–8	5.1	1.0	5	1	3–7	*p* = 0.002
Total	5.7	1.7	5	2	0–8	4.6	1.5	5	2	0–8	*p* < 0.001
Physical pain											
ADDwR	10.2	2.5	10	3.5	3–14	8.7	2.1	9	4	5–12	*p* < 0.001
ADDwoR	12.2	2.0	13	3	8–17	10.4	2.2	10	3	7–17	*p* < 0.001
ADDwoR + OA	13.3	2.5	12.5	3	8–18	11.8	2.0	12	3.25	8–15	*p* = 0.003
Total	11.8	2.7	12	3.5	3–18	10.2	2.4	10	3	5–17	*p* < 0.001
Psychological discomfort											
ADDwR	9.0	2.3	9	3	3–16	6.8	1.5	7	3	3–10	*p* < 0.001
ADDwoR	9.5	2.3	9	3	5–15	7.6	2.1	8	3.25	4–13	*p* < 0.001
ADDwoR + OA	10.1	1.8	10	2.25	7–13	9.1	1.8	8.5	3	4–12	*p* = 0.011
Total	9.5	2.2	9	3	3–16	7.8	2.0	8	3	3–13	*p* < 0.001
Physical disability											
ADDwR	4.1	1.7	4	2.5	0–7	3.3	1.5	3	2.5	0–7	*p* < 0.001
ADDwoR	4.6	1.4	4.5	1	0–7	3.6	1.2	4	1	0–7	*p* < 0.001
ADDwoR + OA	5.1	1.5	5	2	2–8	5.0	1.6	4	2	1–8	*p* = 0.718
Total	4.5	1.6	4	2.5	0–8	3.9	1.6	4	2	0–8	*p* < 0.001
Psychological disability											
ADDwR	9.4	2.9	9	4.5	3–14	7.4	2.5	9	3.5	1–12	*p* < 0.001
ADDwoR	11.2	2.1	11	3	7–16	9.2	1.6	9	2	6–13	*p* < 0.001
ADDwoR + OA	12.4	2.5	13	3	6–18	11.0	2.4	11.5	4	6–15	*p* < 0.001
Total	10.9	2.8	11	4	3–18	9.0	2.6	9	4	1–15	*p* < 0.001
Social disability											
ADDwR	3.6	1.3	3	1.5	2–6	2.8	1.1	3	1	1–5	*p* = 0.001
ADDwoR	4.0	1.6	4	2	1–7	3.3	1.2	4	1.25	1–7	*p* = 0.006
ADDwoR + OA	4.6	1.6	4	2.25	1–8	4.3	1.6	4	3	1–7	*p* = 0.011
Total	4.1	1.5	4	2	1–8	3.4	1.4	3	1.5	1–7	*p* < 0.001
Handicap											
ADDwR	3.8	1.4	4	2	2–7	3.5	1.1	4	1	2–6	*p* = 0.027
ADDwoR	3.9	1.6	4	2	0–7	3.3	1.5	3	2	0–6	*p* = 0.029
ADDwoR + OA	4.8	1.4	5	2	2–7	4.4	1.6	4	2	1–7	*p* = 0.058
Total	4.1	1.5	4	2	0–7	3.7	1.4	4	2	0–7	*p* < 0.001
Global	7.2	3.7	6	6	0–18	6.1	3.2	5	4	0–17	*p* < 0.001

Abbreviations: ADD, anterior disc displacement; wR, with reduction; woR, without reduction; OA, osteoarthritis. The Kolmogorov-Smirnov (K-S) test is used to analyze whether the data conform to the normal distribution. The Wilcoxon signed-rank test was used to assess the significance of the data

### DASS-21

The scores before and after treatment fluctuated between 0 and 19 and 0–17, respectively. The average scores of stress, anxiety, and depression following KDOS treatment were 9.9 ± 3.4, 6.4 ± 3.0, and 6.1 ± 2.9, respectively, which were significantly different from the average scores of 11.4 ± 4.0, 7.2 ± 3.6, and 7.1 ± 3.5 before treatment (*p* < 0.001). The mean scores of the three groups in the three sections were significantly lower than those before treatment (*p* < 0.05). Except for stress, the ADDwoR group demonstrated the highest average score, ADDwoR + OA group had the highest average score in the other two parameters, followed by the ADDwoR group, and the ADDwR group had the lowest score. The mean DASS-21 scores of the three groups are presented in Table 4.

**Table 4:** DASS-21 scores in three groups before and after treatment

Item	Before treatment					After treatment						*p* value
	Mean	SD	Median	Interquartile spacing	Range	Mean	SD	Median	Interquartile spacing		Range	
Stress												
ADDwR	10.6	5.2	11	8.5	0–19	9.2	4.2	10	7	0–17		*p* = 0.009
ADDwoR	12.7	3.4	13	5	2–18	10.4	2.8	10	3.5	3–15		*p* < 0.001
ADDwoR + OA	11.0	2.7	10.5	4.25	7–16	10.2	2.9	9	5	6–15		*p* = 0.021
Total	11.4	4.0	12	5	0–19	9.9	3.4	10	4.5	0–17		*p* < 0.001
Anxiety												
ADDwR	4.8	3.2	4	5	0–11	4.3	2.6	4	4	0–9		*p* = 0.018
ADDwoR	8.0	3.2	8.5	4.25	2–14	8.0	3.2	7	5.25	2–12		*p* < 0.001
ADDwoR + OA	9.2	2.6	9	4	4–15	8.2	2.1	8	2.5	5–13		*P* = 0.030
Total	7.2	3.6	7	6	0–15	6.4	3.0	6	5	0–13		*p* < 0.001
Depression												
ADDwR	4.6	3.1	5	4.5	0–12	4.1	2.4	4	4	0–8		*p* = 0.005
ADDwoR	8.0	3.2	8	2.75	2–14	8.0	3.2	7	3.25	2–11		*p* < 0.001
ADDwoR + OA	9.1	2.7	8.5	2.5	4–15	8.3	2.5	9	3	3–12		*p* = 0.012
Total	7.1	3.5	8	4.5	0–15	6.1	2.9	6	4	0–12		*p* < 0.001

Abbreviations: ADD, anterior disc displacement; wR, with reduction; woR, without reduction; OA, osteoarthritis. The Kolmogorov-Smirnov (K-S) test is used to analyze whether the data conform to the normal distribution. The Wilcoxon signed-rank test was used to assess the significance of the data

### Binary logistic regression

**Fig. 3:**
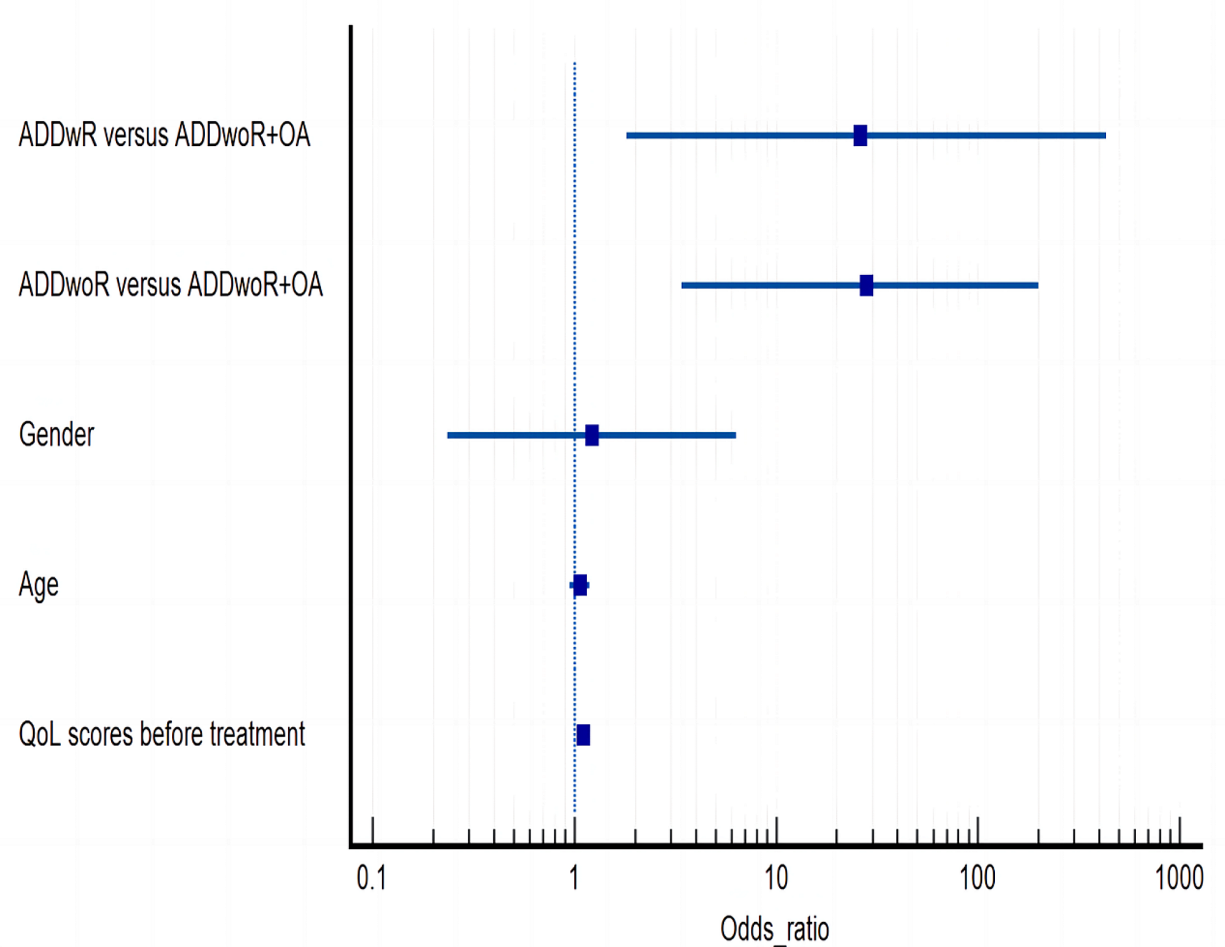
Forest plot of binary logistic regression results. Abbreviations: ADD, anterior disc displacement; wR, with reduction; woR, without reduction; OA, osteoarthritis; QoL, quality of life

Binary logistic regression revealed that (1) compared with the ADDwoR + OA group, the ADDwR and ADDWoR groups were more likely to demonstrate improved clinical outcomes following treatment; (2) patients with low QoL scores before treatment were more likely to demonstrate improved clinical outcomes after treatment; and (3) there was no significant correlation between the improvement in the clinical outcomes and age or sex. The results are presented in Table 5; Fig. 3.

**Table 5:** Results of binary logistic regression

Index	B	S.E.	Wald statistic	df	*p* value	Odds ratio
Wilkes stage						
ADDwR versus ADDwoR + OA	3.360	1.039	9.848	1	0.002	26.041
ADDwoR versus ADDwoR + OA	3.335	1.397	5.703	1	0.017	28.088
Gender	0.203	0.840	0.058	1	0.809	1.225
Age	0.064	0.058	1.236	1	0.266	1.066
QoL scores before treatment	0.097	0.035	7.844	1	0.005	1.102
Constant	-9.034	3.137	8.292	1	0.004	0.000
Hosmer-Lemeshow test					0.165	

Abbreviations: ADD, anterior disc displacement; wR, with reduction; woR, without reduction; OA, osteoarthritis; QoL, quality of life. Binary logistic regression was used to analyze clinical factors associated with improved clinical outcomes

## Discussion

Over the past few years, there has been growing interest in the OHRQoL. Oral diseases can affect various aspects of an individual’s mental and physical health [16]. TMD, which mainly involves the temporomandibular joint and/or masticatory muscles, causes clinical symptoms, such as oral and maxillofacial pain, joint noise, and mandibular movement disorders, which significantly impact the patients’ physical and mental health and QoL [17, 18]. Although the etiology of TMD is vast and various theories exist describing the pathogenesis of TMD, the theory of multifactor co-pathogenesis has been accepted by many researchers. Various treatment methods exist for managing the different pathogeneses of TMD; the chief treatment methods can be classified as reversible treatments, such as physical therapy and drug therapy, and irreversible treatments, such as adjustment of bite and joint surgery [1, 2, 19]. Since the pathogenesis of TMD is still unclear, and long-term research results reveal that the success rate of conservative and non-conservative treatment is similar, clinical treatment mainly comprises non-invasive conservative treatment [2, 20].

Occlusal splint is a conservative treatment modality with wide clinical application and experimentally verified clinical efficacy. The occlusal splint is a removable appliance that can be used to treat oral-maxillary system dysfunction by adjusting the jaw position and occlusal contact, changing the position of the condyle in the articular fossa, and reducing abnormal muscle activity [21, 22]. At present, the stabilization occlusal splint is the most common occlusal splint used for TMD treatment. In recent years, the wide application and rapid development of digital technology in the field of dental prosthetics has resulted in great innovation in traditional design concepts and production modes. For example, Dedem and Türp [23] used computer-aided design for fabricating occlusal splints; intraoral testing demonstrated good retention and stability. However, complete digitalization includes at least three components, namely digital impression technology, digital design, and digital production [24]. KDOS digitizes the entire process and treats TMD by using the balancer to deprogram the muscles to determine the therapeutic jaw position.

Pain, which greatly affects the QoL of patients, is the most common symptom of TMD [25]. The majority of the patients reported pain in the masticatory muscle and / or preauricular region, which was easily exacerbated by chewing or other jaw activity [26]. Chronic pain reportedly has a strong negative impact on the patients’ QoL [9, 27, 28]. In this study, 53 patients (59.6%) had pain symptoms, and the average score in the domain of physical pain was the highest among the seven domains in the OHIP (11.8 ± 2.7). The second highest scoring domain was psychological disability (10.9 ± 2.8), which is supported by a bio-psychosocial model on TMD [29]. After KDOS treatment, the average physical pain score of the patients significantly reduced (10.2 ± 2.4, *p* < 0.001), and the improvement rate reached 69.8%, which was similar to the improvement rate (71.6%) in a previous KDOS study by Hua et al. [13]. Consistent with their study, the pain improvement rate in the ADDwoR + OA group was the lowest among the three Wilkes stage groups, which may be related to greater tissue damage in the disease. However, according to Pficer et al.‘s 2017 meta-analysis [30], the effect of the stabilization occlusal splint on the pain outcomes was significantly better than that of the control group in the short-term, while this effect gradually disappeared in the long-term study; therefore, a longer follow-up period is warranted to support the results of our study. Furthermore, in this study, the JNS was separated from the Friction index, and a quantitative index was applied to ensure objectivity of the compared result. Joint noise and MMO also significantly improved after treatment, with the joint noise improvement rate reaching 80.4%. Tecco et al. [31] demonstrated that more than 50% of the patients still experienced persistent joint noise three months after treatment, regardless of whether they were treated with a repositioning or stabilization occlusal splint, indicating that joint noise is likely to have a persistent impact on the patients’ QoL. Previous studies have demonstrated that both the MMO and OHIP scores improved after occlusal splint treatment [32, 33]. Our study supported this view; all seven domains of the OHIP and global mean scores significantly improved following KDOS treatment.

Studies have demonstrated that TMD can hinder daily, social, and family activities, which can lead to poor mental and emotional states [27, 34]. This study reached the same conclusion; in the DASS-21, the average scores for stress, anxiety, and depression exceeded the normal range (11.4 ± 4.0, 7.2 ± 3.6, 7.1 ± 3.5, respectively). A large proportion of patients with TMD report difficulty falling asleep or staying asleep, indicating that poor sleep quality in patients with TMD is an important issue, as physical and mental health is associated with effective sleep and contributes to good QoL [35, 36]. While sleep quality is closely related to stress levels, the average stress score in this study was the highest among the three items; however, no relevant evidence exists for the effect of sleep on the QoL. Additionally, since emotional stress caused by TMD can lead to anxiety and depression, most patients require psychological assistance [37]. After KDOS treatment, the patients demonstrated significant improvement in their average scores concerning stress, anxiety and depression (9.9 ± 3.4, 6.4 ± 3.0, 6.1 ± 2.9, respectively; *p* < 0.001). Pficer et al. [30] demonstrated a similar result for the stabilization occlusal splint, which supports the findings of this study.

The binary logistic regression analysis revealed that patients with low QoL scores before treatment and those in the ADDwR and ADDwoR groups were likely to demonstrate a higher rate of improvement in the clinical symptoms following treatment with KDOS, which is similar to the results of Hua [13] and Clark et al. [38] GHE et al. [39] also reported that patients with joint pain or OA had lower QoL than those with myofascial pain or disc displacement. The present study concluded that the improvement rate of clinical symptoms was not associated with the age and sex of the patients; moreover, Emshoff et al. [40] did not find any influence of age on the prognosis of stabilization occlusal splint treatment; however, Hua et al. [13] demonstrated that the older the patient, the higher the possibility of improvement, which may be attributed to the difference in sample size and population characteristics.

In this study, patients with TMD demonstrated significant improvement in their clinical symptoms and QoL scores following treatment with KDOS; however, some studies have shown that occlusal splints and other treatments (such as acupuncture, counseling, and masticatory muscle exercises) have little or no significant benefit in alleviating symptoms [41–43]. Additionally, some studies have highlighted the limited efficacy of occlusal splint for TMD patients with OA. However, the application of arthroscopic surgery and/or platelet-rich plasma has been shown to significantly ameliorate clinical symptoms in OA patients, including pain and mouth opening [44, 45]. Furthermore, our study’s findings revealed no significant improvement in most items related to OHRQoL scores following the use of KDOS in the ADDWoR + OA group. Thus, further research should focus on randomized controlled trials comparing KDOS with other treatments.

## Conclusions

KDOS can improve the clinical symptoms and QoL of patients with TMD. Clinical improvement is related to the severity of the disease and the level of QoL before treatment.

## Data Availability

The datasets used and/or analysed during the current study are available from the corresponding author on reasonable request.

## Abbreviations

TMDs: Temporomandibular disorders
OHRQoL: Oral health-related quality of life
KDOS: Kovacs digital occlusal splint
ADDwR: Anterior disc displacement with reduction
ADDwoR: Anterior disc displacement without reduction
OA: Osteoarthritis
MMO: Maximal mouth opening
Pain-VAS: Pain-visual analog scale
JNS: Joint noise score
DASS-21: Depression, Anxiety, and Stress Scale-21
OHIP-TMD: Oral Health Impact Profile for TMDs
